# Environmental Life Cycle Assessment of a Novel Hemp-Based Building Material

**DOI:** 10.3390/ma16227208

**Published:** 2023-11-17

**Authors:** Daniela Rivas-Aybar, Michele John, Wahidul Biswas

**Affiliations:** Sustainable Engineering Group, School of Civil and Mechanical Engineering, Curtin University, Bentley, WA 6102, Australia; d.rivasaybar@postgrad.curtin.edu.au (D.R.-A.); m.rosano@curtin.edu.au (M.J.)

**Keywords:** biomaterials, hemp-based materials, life cycle assessment

## Abstract

The global construction sector contributes a significant share of total greenhouse gas (GHG) emissions. In Australia, infrastructure activity alone generates 18% of the GHG emissions. The use of low-embodied carbon building materials is crucial to decarbonise the construction sector and fulfil national and international climate goals. Industrial hemp (*Cannabis sativa* L.) is a promising feedstock for low-carbon construction materials because of its carbon sequestration capacity, fast-growing cycles, and technical functionality comparable to traditional materials. This study utilised the life cycle assessment (LCA) guideline ISO 14040:2006 to estimate the carbon footprint (CF) of hemp-based building materials in Western Australia capturing region-specific variations in terms of inputs, soil, productivity, and energy mix. The functional unit was 1 m^2^ of a hemp-based board, and the system boundary was cradle-to-gate, i.e., pre-farm, on-farm, and post-farm activities. The CF of 1 m^2^ of hemp-based board was estimated to be −2.302 kg CO_2_ eq. Electricity from the public grid for bio-based binder production during the post-farm stage was the main contributor to total CO_2_ eq emissions (26%), followed by urea production (14%) during the pre-farm stage. Overall, the use of electricity from the public grid during the post-farm stage accounted for 45% of total emissions. Sensitivity analysis showed that the CF of hemp-based boards was highly sensitive to the source of energy; i.e., total replacement of the public grid by solar power decreased the CF by 164% (−2.30 to −6.07 kg CO_2_ eq). The results suggested that hemp-based boards exhibit lower embodied GHG emissions compared to traditional materials, such as gypsum plasterboards.

## 1. Introduction

Climate change is one of the greatest challenges for human development [1]. The sharp increase in heat-trapping gases, i.e., greenhouse gases (GHG), in the atmosphere has increased surface temperatures by 1.1 °C above pre-industrial levels [2]. Current mitigation policies aim to limit the global temperature increase to 1.5 °C by 2100 [3]. This monumental task requires anthropic intervention to actively sequester GHG from the atmosphere to achieve a ‘net-zero’ balance between GHG emission and removal.

More than one third of global energy-related carbon dioxide equivalent (CO_2_ eq) emissions originate from the building and construction sector [4]. In Australia, this sector alone contributes to 18% of the country’s total GHG emissions [5]. Aligned with Australia’s climate goal of achieving net-zero GHG emissions by 2050 [6], there is an urge to decarbonise the built environment.

In recent years, efforts to decarbonise infrastructure activities have primarily focused on reducing GHG emissions during the operational phase (i.e., heating, cooling, ventilation, lighting, and hot water supply), with little consideration for embodied carbon (i.e., construction materials manufacturing, materials transportation, construction, and demolition) [7]. In Australia, as the operation stage of buildings decreases associated GHG emissions due to the integration of renewable energy, embodied carbon assumes a greater share of the building’s emissions budget [8]. Therefore, it is urgent to implement measures to reduce embodied carbon across the entire building life cycle [9]. To this extent, the life cycle assessment (LCA) method has been extensively employed to identify materials or processes that contribute the most to GHG emissions (hotspots) over the building’s lifespan.

LCA-based studies emphasise the critical role of construction materials in decarbonising the sector [7,10]. Plant-based materials have gained attention for their CO_2_ sequestration capacity and technical functionality, which can help reduce the GHG emissions of buildings [11]. Among various plants suitable for manufacturing building elements, industrial hemp (*Cannabis sativa* L.) stands out as a promising solution due to its fast growth cycles with relatively low fertiliser and pesticide requirements, thermal and acoustic insulation functionality, and lower embodied carbon compared to traditional mineral-based materials [12].

Furthermore, hemp hurds, which constitute the woody core of the hemp stalk, are the primary component of lime–hemp concrete (LHC), one of the most extensively studied plant-based construction materials [13,14]. LHC consists of hemp hurds, lime-based binder, and water [15,16]. There is growing interest in quantifying the environmental benefits derived from using hurds in construction, as evidenced by the increasing body of LCA studies assessing its carbon footprint (CF), with most research conducted in Europe [17,18,19,20,21]. In these studies, the CF of hurds was found to vary across regions (Table 1). Research has also reported that the amount of CO_2_ sequestered through photosynthesis during plant growth, also known as biogenic carbon [15,22], typically exceeds the CO_2_ eq released during its life cycle when excluding end-of-life, i.e., the production of agricultural inputs, machinery, cultivation, transportation, and processing. Thus, various authors have expressed the CF of hurds with a negative value as displayed in Table 1.

Climatic conditions, soil properties, the type of production system, and the electricity mix are factors that significantly influence the agricultural CF [23]. For example, emissions of nitrous oxide (N_2_O) (273 times more potent than CO_2_ [22]) resulting from nitrogen (N) fertiliser application are highly influenced by climatic conditions [24] and soil characteristics [25,26,27]. Consequently, the CF of hurds produced in Australia, where the hemp industry is rapidly expanding [28], may differ markedly from production in other parts of the world.

**Table 1:** CF in kg CO_2_ eq per kg of hurds reported in previous LCA studies.

Location of the Study	CF kg CO_2_ eq/kg Hurds	Reference
United Kingdom	−1.335	[21]
Italy	−1.730 to −1.750	[19]
France	−1.550 to −1.630	[20]
France	−0.315 to −0.558	[17]
Serbia	−1.182 to −1.380	[18]

Similarly to its main component, the CF of LHC varies across regions [12] (Table 2). Most LCA studies have also reported that LHC has the potential to be a carbon negative material [18,20,21,29,30,31], and highlight the production of lime-based binders as a hotspot during its life cycle [9,32,33]. Moreover, when large amounts of lime-based binders are used, the resultant material tends to be carbon positive, meaning that more CO_2_ eq is released than sequestered [18]. Lime is produced from limestone, which undergoes a calcination process at a temperature between 900 and 950 °C [16,34]. The process requires high amounts of energy (usually non-renewable) and thus accumulates a significant amount of embodied carbon [35]. The amount of CO_2_ released during calcination is approximately 600 g per kg of lime [29]. The lime production process also has other considerable environmental impacts, particularly with air pollution [14]. Thus, it is necessary to find more environmentally friendly alternatives to this traditional binder, without affecting the technical performance of LHC.

Various studies have reported that LHC exhibits thermal conductivity between 0.05 and 0.12 W/mK, moisture buffer values higher than 2 g/(m^2^% RH) [36], and acoustic absorption coefficients that range from 0.24 to 0.53 [37]. Therefore, it presents functional thermal, hygric, and acoustic properties. Moreover, LHC properties contribute to reducing operational energy consumption while maintaining indoor comfort offering an alternative to traditional materials [29]. However, while LHC has lower strength compared to conventional concrete [32], some scholars have considered that this material is most suitable to replace gypsum plasterboards [38].

Traditional plasterboards consist of a dense gypsum core protected on its sides by a cellulose layer [39]. The manufacturing process begins with extracting of gypsum rocks from quarries and transporting them to a processing facility, where they are crushed and ground into a powder, which is then calcinated at 160 °C, resulting in the accumulation of significant GHG emissions [13,40]. In fact, plasterboards can account for 0.4% of the buildings’ material stock in major Australian cities [41], and has been identified as one of the top five building materials in terms of embodied carbon and energy by the Green Building Council of Australia [8].

This paper aims to evaluate the life cycle environmental impact of a novel board that uses a mixture of hemp hurds and a bio-based binder from an Australian context, capturing the region-specific variation in term of inputs, soil, productivity, and energy mix. The research applies the LCA methodology to estimate the carbon footprint (CF) of this innovative material, as it has the potential to help decarbonise the Australian construction sector [12]. To determine the environmental benefits of these boards, the results are compared with other hemp-based materials and gypsum plasterboards. The research further performs a hotspot analysis to identify the inputs and processes contributing the largest share of GHG emissions and find improvement strategies to mitigate them.

## 2. Materials and Methods

The LCA methodology, following ISO 14040:2006 standards [42,43] was applied to calculate the CF of hemp-based boards developed in Western Australia (WA). These boards are composed of a mixture of hemp hurds and a bio-based binder. Previous research have found that the production of similar materials results in various environmental impacts, including global warming, land use change, eco-toxicity, and eutrophication [44]. However, this single-focused LCA only considers global warming impact aligning with Australia’s commitment for meeting urgent decarbonisation targets [6]. The project’s funders, namely the Food, Fibre, and Land International (FFLI) group and MIRRECO^®^, have also expressed interest in estimating the CF of their hemp-based products.

Like Finkbeiner [45], CF in this research is considered in terms of an LCA, with the limited focus on one impact category only, i.e., the global warming impact. All methodological requirements and principles of the LCA can be used to determine CF, as evidenced by local and international research in the literature [5,18,26,35,46,47]. It is worth mentioning that the CF of hemp hurds and hemp-based building materials has been previously estimated using the LCA approach (see Table 1 and Table 2). This tool facilitates a comprehensive appraisal of the system’s hotspots, enabling end-users to formulate strategies for improvement. The ISO 14040:2006 organises the method into four distinct phases: (i) definition of the goal and scope; (ii) life cycle inventory analysis (LCI); (iii) impact assessment (LCIA); and (iv) interpretation [42,43]. The fourth phase, interpretation, is presented in the Results and Discussion section.

### 2.1. Goal and Scope Definition

The goal of the study was to estimate the CF (expressed in CO_2_ eq) of hemp-based boards taking into account region-specific variables for Western Australia The study applied the GWP 100a method [48], which has been the preferred methodology used in similar studies [18,20,21,29,30,31]. To determine the environmental benefit of hemp hurds and hemp-based boards produced in Australia, the results were compared with the LCAs of hemp hurds and hemp-based construction materials and traditional materials, i.e., gypsum plasterboards [38], that followed the GWP 100a method. In addition, the hotspots identified were examined through sensitivity analyses.

**Figure 1:**
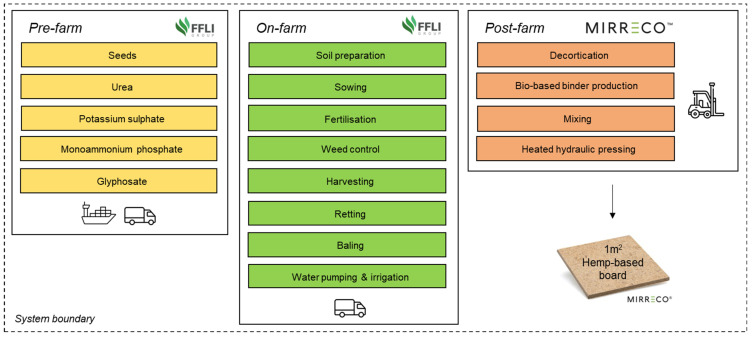
System boundary for conducting the life cycle assessment of 1 m^2^ of hemp-based board.

The functional unit (FU) was one square meter (1 m^2^) of hemp-based board sizing 100 cm × 100 cm × 1.25 cm with a hurds-to-binder mass ratio of 2:1. This material has been developed by MIRRECO^®^. The system boundary studied includes cradle-to-gate stages, involving pre-farm, on-farm, and post-farm activities (Figure 1) as follows:

- Pre-farm: production of agricultural inputs and its transport to paddock (plot of land on a farm).
- On-farm: operation of farming machinery, transportation of hemp bales from paddock to processing plant, soil emissions from N fertilisation, and biogenic carbon sequestration.
- Post-farm: indoor transportation of raw materials, decortication, bio-based binder production, mixing of hurds and binder, and heated hydraulic pressing.

It is worth noting that the CO_2_ stored in the hemp-based board will ultimately be released into the atmosphere at the end-of-life stage as a result of its transformation or degradation [49]. In the LCAs conducted to date [15,17,30], the CO_2_ is considered to be trapped in hemp hurds and other bio-based materials prior to their disposal or degradation period. The current LCA follows the same approach as its system boundary is limited to cradle-to-gate stages.

### 2.2. Life Cycle Inventory Analysis (LCI)

The LCI comprised the data collection for the quantification of relevant inputs and outputs within the system boundary of 1 m^2^ of hemp-based board. Table 3 summarises the inventory inputs needed to produce the FU, which was determined through a mass balance.

#### 2.2.1. Pre-Farm Stage

Primary data for the pre-farm stage were acquired through interviews with hemp growers from the FFLI and expert advisors from the Department of Primary Industries and Regional Development (DPIRD) in Perth. These interviews were conducted during March and May 2023 and involved site visits to a hemp plantation in Kaloorup (−33°45′ S, 115°14′ E), situated in the South West of WA. The soil type prevalent in this area was sandy loam. This paddock was established in 2022; therefore, the gathered information corresponded to that same year. The data collected involved a comprehensive inventory of the quantities and sources of the inputs required to produce 1 hectare of hemp biomass (hurds and fibres), i.e., seeds, fertilisers, and herbicides.

Table 4 summarises the transportation mode and average distances assumed for conveying the inputs from the manufacturing site to the paddock. The transportation of seeds, primarily cultivated on a small scale in Esperance (WA), was assumed to employ a 3.5-tonne truck. For inputs manufactured overseas, such as potassium sulphate and glyphosate, transportation via freight ship and 20-tonne articulated trucks was assumed. A 20-tonne articulated truck was also assumed for the transport of other inputs within the country, given its widespread usage across regional Australia [26]. Distances were calculated under the assumption that the farm is located in Kaloorup. Within this region, the preferred hemp variety for biomass production is Frog 1. The sowing usually starts in spring and harvest takes place in autumn (Telfer, D., DPIRD representative, pers. comm., 7 July 2023).

**Table 4:** Assumptions about transport of input materials to the farm.

Inputs	Transportation Mode		Average Distance (km)	
	Sea	Land	Sea	Land
Seeds	-	3.5- to 16-tonne truck	-	716
Urea	-	20-tonne articulated truck	-	1778
Potassium sulphate	Freight ship	20-tonne articulated truck	7477	363
Monoammonium phosphate (MAP)	-	20-tonne articulated truck	-	218
Glyphosate	Freight ship	20-tonne articulated truck	17,314	3720

According to the information provided in May 2023, the average yield for good conditions in Kaloorup was 10 tonnes/ha of hemp biomass, which comprised 7 tonnes of hurds, 2.5 tonnes of fibres, and 0.5 tonnes of dust. The seeding rate of 30 kg/ha was recommended to obtain optimum biomass yield. The soil in WA generally requires the application of 114 kg of N, 45 kg of P_2_O_5_, and 60 kg of K_2_O per hectare. The amount of herbicide considered was 1 L of glyphosate/ha. The application of pesticides was not necessary for the referenced paddock.

#### 2.2.2. On-Farm Stage

Similar to the pre-farm stage, inventory data were gathered through interviews with growers from the FFLI group. This stage involved the use of farming machinery, including tractors equipped with various attachments, such as rippers, seeders, sprayers, harvesters, harrows, and balers, as well as a water pump and a centre pivot. The machinery is used to perform the following farming operations: soil preparation, sowing, fertilisation, weed control, harvesting, retting, baling, water pumping, and irrigation, respectively. Most of the machinery is standard and adaptable for use with other annual crops commonly grown in the region, such as wheat. The only machinery exclusive to hemp cultivation was the harvester (specifically, the hemp cutter Laumetris KP-4), with technical specifications sourced directly from the manufacturer, Forever Green. This stage also considered the transportation of hemp bales from the farm to the processing facility, with the assumption that the bales were transported an average distance of 243 km using a 20-tonne articulated truck.

Soil CO_2_ and N_2_O emissions derived from N fertiliser application (urea and MAP) and CO_2_ sequestered during plant growth were also considered at this stage.

#### 2.2.3. Post-Farm Stage

Input data for the post-farm stage were obtained from the representatives of MIRRECO^®^. When necessary, complementary data were sourced from credible sources, i.e., technical specifications from industrial machinery manufacturers and the scholarly literature, as specified in Table 3. Inventory data included diesel and electricity demand for industrial equipment to process and manufacture a 1 m^2^ hemp-based board, i.e., indoor transport of raw materials, decortication, bio-based binder production, mixing of hurds and binder, and hydraulic pressing. A 1 m^2^ hemp-based board consists of 5.154 kg of hurds and 2.577 kg of binder.

During the decortication process, hemp stalks undergo crushing, leading to the separation of hurds from fibres. Following decortication, three co-products are obtained: fibres, hurds, and dust. Fibres and dust are transported and stored for different purposes not considered in this inventory. It is noteworthy that this study regards hurds as the primary product of hemp cultivation unlike other studies where the fibre is recognised as the primary product [31]. This choice was based on local demand, as hemp fibres have limited significance in the region [12]

Subsequently, the hurds and bio-based binder are mixed in a 2:1 ratio and then transported for thermocompression using a heated hydraulic press to produce the final product.

### 2.3. Life Cycle Impact Assessment (LCIA)

The SimaPro 9 software package was employed to convert LCI results into CF using the GWP 100a method. This software facilitated the linkage of most inventory data with the Australian National Life Cycle Inventory Database (AusLCI), which draws from Australian sources [52]. However, certain inputs and outputs from the inventory were absent in AusLCI including hemp seed production, soil CO_2_ and N_2_O emissions from the application of N fertilisers and the biogenic uptake of hemp. To address these gaps, new databases were created within the software, guided by the following considerations and assumptions:

- Hemp seed production: Information collected during interviews with farmers was utilised to construct this process since inputs and machinery for hemp seed production align with those for hemp biomass production (Edkins, R., hemp grower, pers. comm., 18 April 2023).
- Direct CO_2_ emissions from urea application: These emissions due to urea hydrolysis were estimated using a CO_2_-C emission factor (EF, the percentage of urea that is lost as CO_2_-C) of 20%. This is a default value proposed by the Intergovernmental Panel on Climate Change (IPCC) [53]. This value was applied due to the absence of specific data for Kaloorup. CO_2_-C emissions were multiplied by 44/12 to determine CO_2_ emissions.
- Direct N_2_O emissions from N fertilisation: The estimations about the fraction of the N fertiliser that is transformed and emitted as N_2_O emissions have a significant effect on the CF of agricultural products grown in WA’s South West, as evidenced in the literature [26,47,54]. Moreover, various regional studies have measured N_2_O emissions in situ instead of relying on default values to calculate them [25,54,55,56]. However, this study was limited to estimating direct N_2_O emissions using scholarly sources because specific data were not available for the study site. Accordingly, the EF for direct N_2_O emissions was sourced from a meta-analysis conducted by Cayuela, Aguilera [24], which included prior regional studies [25,54,55,56]. The meta-analysis suggests that 0.63% of the N input is lost as N_2_O-N emissions in WA’s South West soils under irrigation. N_2_O-N emissions were multiplied by 44/28 to determine N_2_O emissions.
- Indirect N_2_O emissions from N fertilisation: these emissions correspond to the portion of the N fertiliser that is lost through leaching and volatilisation. According to the IPCC, N leaching only occurs when the evapotranspiration to annual precipitation ratio is between 0.8 and 1.8 [53]. This ratio was 2.3 in 2022 for the study area, and thus, emissions from leaching were considered to be zero. For N volatilisation, emissions were estimated according to the IPCC default EF, which assumes that 10% of N fertiliser is lost as NH_3_, with 1% of the NH_3_ then emitted as N_2_O-N following atmospheric deposition. IPCC default values were used since regional-specific data were not available.
- Biogenic carbon uptake: to the best of the authors’ knowledge, there are no studies that have estimated the biogenic uptake of hemp production in Australia. Therefore, the study used a sequestration factor obtained from an Australian Parliament House report, which estimated 1.37 tonnes of CO_2_ is absorbed per tonne of hemp stalks, based on data from the United Kingdom (UK) [57].

#### 2.3.1. Allocation Method

An allocation method was adopted to differentiate the CF of hurds, fibres and dust, which are the co-products obtained from hemp biomass. These co-products account for 70%, 25%, and 5% of the total biomass, respectively. Allocation methods are generally based on mass or economic values which involve using the weight and prices of the co-products per unit of product [58]. This study considered the appropriate use of a mass allocation approach because the co-products involved do not have stable prices in the local market (Campbell, D., hemp grower, pers. Comm., 7 March 2023), which can affect the validity of the LCA results [59]. Accordingly, the CF of hurds production is allocated by mass at 70% of the total CF of hemp biomass production.

#### 2.3.2. Monte Carlo Simulations (Uncertainty Analysis)

There may be uncertainties associated with the inventory data which can vary according to various factors aforementioned, such as sources, quality, and the availability of information. These uncertainties can affect the LCA outputs, and therefore, to estimate the uncertainty of the life cycle results, Monte Carlo simulations (MCS) were conducted in the SimaPro 9.2 software package for 1000 iterations with a confidence level of 95%.

## 3. Results and Discussion

### 3.1. Monte Carlo Simulations Results

**Figure 2:**
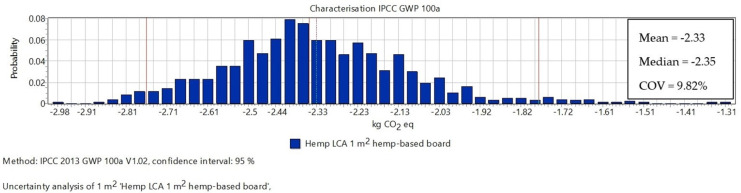
Uncertainty analysis of 1 m^2^ hemp-based board using MCS.

As displayed in Figure 2, the mean value of CF of the overall scheme was −2.33 kg CO_2_ eq/m^2^ (Figure 2). The coefficient of variation (COV) estimated was 9.82% of the mean value, which demonstrates that the results of the LCA study are acceptable [60].

### 3.2. Life Cycle Interpretation

The CF of 1 m^2^ of hemp-based board was estimated to be −2.302 kg CO_2_ eq, meaning that the total CO_2_ eq emitted across its life cycle (8.290 kg CO_2_ eq/m^2^) was lower than the CO_2_ captured during hemp growth (10.592 kg CO_2_ eq/m^2^). The CF of hemp-based boards was divided into two different phases: CF of hemp hurds, i.e., from paddock to hurds, and the CF of hemp-based boards, i.e., from paddock to board, which has been discussed separately in the following sections:

#### 3.2.1. Carbon Footprint of Hemp Hurds

**Figure 3:**
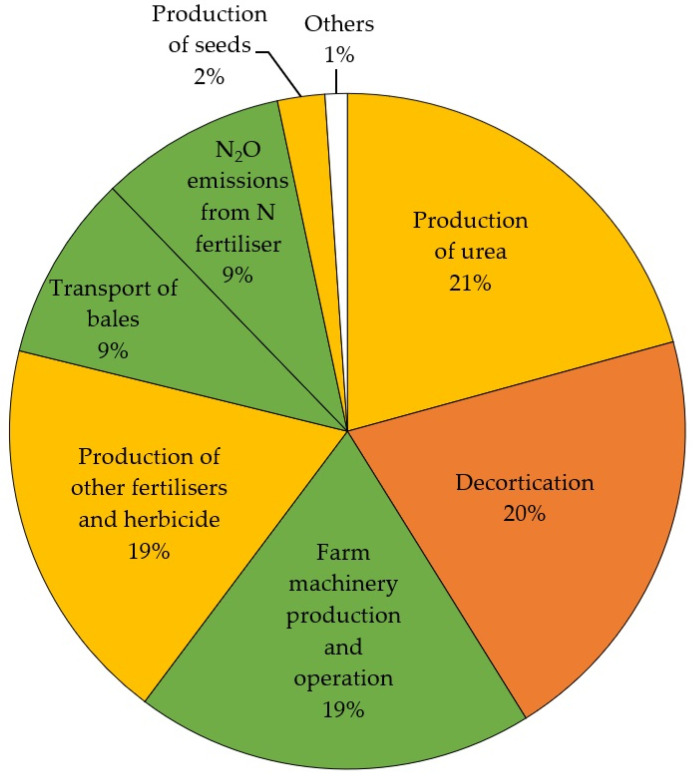
Percentage contributions of CO_2_ eq emissions in terms of inputs and outputs for hurds production excluding the biogenic uptake.

The CF of 1 kg of hemp hurds produced in Kaloorup was estimated to be −1.031 kg CO_2_ eq. The CF of hurds is the balance between the CO_2_ uptake during the on-farm stage and the CO_2_ eq emitted during pre-farm, on-farm, and post-farm stages until the decortication process. Figure 3 presents the percentage contributions of CO_2_ eq emissions in terms of inputs and outputs of hurds production excluding the biogenic uptake. As can be seen in Figure 3, the main contributor to global warming impact was the production of urea (21%), which was the richest N fertiliser. These results are consistent with those reported in similar studies as shown in Table 5.

Table 5 presents the results of the current study along with the outcomes of research that have dealt with CO_2_eq emissions and CO_2_ uptake per 1 kg of hemp hurds. The data that were not directly mentioned in the studies have been extracted from tables and figures. When necessary, the CF of 1 kg of hurds has been estimated by counterbalancing CO_2_ eq emissions and CO_2_ biogenic uptake. These data points have been marked with an approximation symbol (≈).

The literature review suggests that the CF of hemp hurds is affected by myriad factors including site-specific parameters such as yield, agricultural inputs requirements, and the biogenic uptake [17,18,20]. The CF is also influenced by methodological aspects such as the choice of the allocation method (e.g., mass, or economic), which largely depends on the co-products considered in the analysis, e.g., fibres, dust, and seeds.

Spatial variability affects the CF of hurds as the inventories (i.e., yields, fertilisers, pesticides, herbicides, and irrigation), vary considerably across regions, even for the same country. For instance, a study conducted in Italy reported a biomass yield of 15 tonnes/ha [19], 1.5 times more than the productivity considered for this Australian study, i.e., 10 tonnes/ha, and almost double than the yields estimated for France, i.e., 8 and 9 tonnes/ha [17,20]. The location also determines the input requirements; for example, the use of pesticides is not needed in Kaloorup, which is consistent with other study sites [18,19,20]. Conversely, the use of herbicides and irrigation systems are necessary in this location, thus differing from other regions where these inputs are not required [17,18,20].

Similarly, the biogenic uptake of hemp is highly influenced by climatic conditions and thus varies across locations [18]. Bošković and Radivojević [18] examined the effect of using different biogenic uptake values on the CF of hemp-based materials. The study used three sequestration factors obtained from the regional literature, i.e., 1.448, 1.349, and 1.547 kg CO_2_ eq, which correspond to the baseline, pessimistic and optimistic scenarios, respectively. The use of the optimistic uptake value reduced the CF of hurds by 14% compared to the use of a pessimistic value, which suggested that the CF is affected considerably by the choice of the uptake value. The authors concluded that high CO_2_ biogenic uptake can outweigh CO_2_ eq emissions during the life cycle of hemp products. In the case of Australia, there are no sufficient credible sources to obtain different uptake values to conduct a sensitivity analysis for the current study and assess its influence on the CF. Nevertheless, sensitivity analyses were conducted to assess other variables that can potentially affect the CF of hemp hurds for this study.

**Table 5:** Comparison of the results with previous LCA studies assessing hemp hurds for construction materials.

Location of the Study	EF for Direct N_2_O-N Emissions from NFertilisers	Hotspot	Co- Products	Allocation Method and Percentage Allocated for Hurds	Hemp Hurds (kg CO_2_ eq/kg of Hurds)		CF (kg CO_2_ eq/kg of Hurds)	Sensitivity Analysis	Ref.
					Emissions	Uptake			
The existing literature									
UK	Not specified	Fertiliser	f, d	Not specified	≈ 0.192	1.527	≈−1.335	-	[21]
Italy	1.70%	Fertiliser	f, d	Mass 75%	≈ 0.100	1.830	−1.730	-	[19]
				Economic 61%	≈ 0.080	1.830	−1.750		
West France	1.25% *	N fertiliser	f, d, s	Mass 47%	≈ 0.290	1.840	−1.550	-	[20]
				Economic 32%	≈ 0.210	1.840	−1.630		
Vendée (France)	1.25% *	N fertiliser	f, s	Mass 56%	0.975	1.290	≈−0.315	Baseline	[17]
					0.853	1.290	≈−0.437	Use of compost, 50%	
					0.886	1.290	≈−0.404	Use of compost, 75%	
					0.732	1.290	≈−0.558	Use of compost, 100%	
Serbia	1.25% *	Not specified	f, d	Mass 60%	0.167	1.448	≈−1.281	Baseline	[18]
					0.167	1.349	≈−1.182	Pessimistic	
					0.167	1.547	≈−1.380	Optimistic	
Current study									
Kaloorup (Australia)	0.63%	N fertiliser	f, d	Mass 70%	0.339	1.37	−1.031	Baseline	
	0.06%				0.312	1.37	−1.058	EF, local literature [54]	
	1.00% *				0.357	1.37	−1.013	EF, IPCC default value	
	0.63%			Economic 49%	0.237	1.37	−1.133	Economic allocation	

* IPCC default EF for direct N_2_O-N emissions from N fertilisers. Abbreviations of hemp co-products in this table: fibres (f); dust (d); seeds (s).

##### Sensitivity Analyses

- Direct N_2_O emissions from N fertiliser application

Direct N_2_O emissions is another factor that is largely influenced by climatic [24] and soil characteristics [25,26,27], but usually not considered in sensitivity analyses of hemp studies. The choice of the method for estimating N_2_O emissions, i.e., measurements in situ or calculations based on IPCC default values, can significantly affect the CF of agricultural products grown in WA’s South West, as evidenced in various regional studies [26,47,54]. Biswas, Barton [26], for instance, reported that the use of on-site measurements of N_2_O emissions decreased the CF of wheat by 38% compared with that estimated using IPCC values.

In this view, a sensitivity analysis was conducted to examine the effects of varying the estimations of N_2_O emissions on the CF of hurds (Table 5). For this purpose, two alternative EFs for N_2_O emissions were applied, namely 0.06% and 1%, corresponding to a regional study [54] and the IPCC default values [53], respectively. The analysis reported that the use of a regional EF reduced the CF by 4% compared with that calculated using a default value. These findings suggest that the choice of the EF for estimating direct N_2_O emissions have a clear influence on the CF of hurds. In addition, future research should consider on-site measurements of N_2_O emissions because of its effect on the CF of agricultural products in the region.

- Allocation method

Another methodological aspect that can affect the CF of hemp hurds is the allocation method. This aspect was considered by Zampori, Dotelli [19], who estimated a CF of hemp hurds of −1.730 kg CO_2_ eq when using the mass allocation method and −1.750 kg CO_2_ eq when using the economic allocation approach. The difference between these two CF values is approximately 1%, indicating that the choice of the allocation method had a minimal impact on the CF in that particular study. In contrast, Heidari, Lawrence [20] reported a more significant difference in CF values, with a 5% variation when using mass and economic allocation methods, suggesting higher sensitivity to allocation method variation in their research. To assess the sensitivity of the CF for the present study, the economic allocation method was tested using March 2023 prices. Interestingly, the use of the economic allocation method reduced the CF by 9%. The latter results are in line with previous LCA studies that suggested the choice of the allocation method has a considerable effect on various environmental impacts of hemp-based products, including global warming impact [58,61].

##### Mitigation Strategy: Reduction in Synthetic N Fertiliser

The production of synthetic N fertilisers, e.g., urea and ammonium sulphate, have been identified as the hotspot in most studies [17,19,20,21] including the current research (Table 5). In that view, some authors have explored mitigation strategies focused on decreasing the amount of synthetic N input. Scrucca, Ingrao [17], for example, reported that substituting ammonium sulphate with compost in varying ratios of 50%, 75%, and 100% led to reductions in CO_2_ eq emissions from hurds production by 13%, 9%, and 25%, respectively.

In a similar vein, this article examined the implementation of a rotation system involving a legume and hemp as a strategy to enhance N fixation in the soil during legume growth and reduce the quantity of urea applied in subsequent hemp cultivation. This mitigation strategy was grounded on a study that reported that the installation of a two-year lupin-wheat rotation decreased the CF of wheat by 35% in WA’s South West [62]. This reduction occurred due to an increment of N in the soil during legume growth which allowed the reduction of 30 kg of N/ha (65.2 kg of urea/ha) for wheat farming. This reduction not only curbed GHG emissions associated with urea manufacturing but also from its associated soil emissions.

Drawing from supplementary data provided by the authors [62], the mitigation strategy was conducted assuming a two-year lupin–hemp rotation. The analysis concluded that the introduction of lupin before hemp in a rotation system can reduce the CF of hemp hurds by 8%, i.e., from −1.031 kg CO_2_ eq to −0.955 kg CO_2_ eq. However, further research is needed to determine if this mitigation strategy is economically viable to promote adoption among hemp farmers.

#### 3.2.2. Carbon Footprint of Hemp-Based Boards

**Figure 4:**
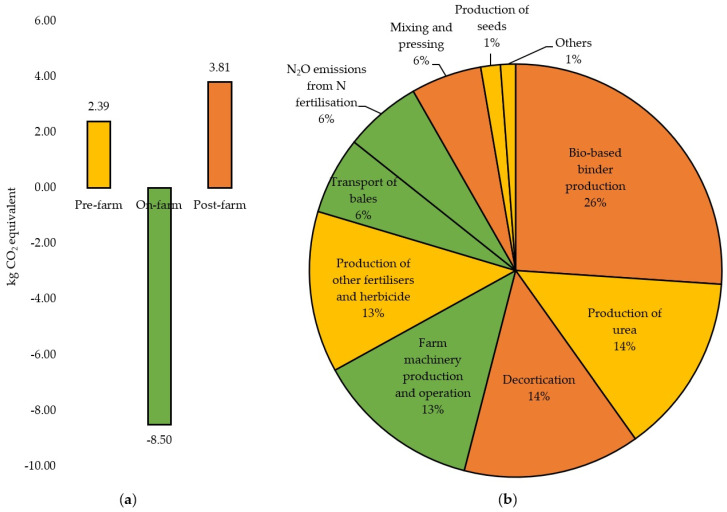
(**a**) CO_2_ eq emissions (kg) generated during pre-farm (dark yellow), on-farm (green), and post-farm stages (orange) for 1 m^2^ of hemp-based board including the biogenic carbon. (**b**) Percentage contributions of CO_2_ eq emissions in terms of inputs and outputs for hemp-based board production excluding the biogenic uptake.

As previously mentioned, the estimated CF of 1 m^2^ of hemp-based board production is −2.302 kg CO_2_ eq, including biogenic uptake. Figure 4a summarises the contribution of each stage to the CF, while Figure 4b presents the percentage contributions of CO_2_ eq emissions in terms of inputs and outputs, excluding biogenic uptake. As seen in Figure 4b, the bio-based binder production during the post-farm stage has the highest impact on CO_2_eq emissions, accounting for over one fourth of the total emissions (26%). Other important carbon sources were the production of urea during the pre-farm stage (14%) and the decortication process during the post-farm stage (14%). Overall, the use of electricity sourced from the South West Interconnected System (SWIS) grid during the post-farm stage, i.e., decortication, binder production, mixing, and heated hydraulic pressing, contributed to 45% of total CO_2_ eq emissions. This is mainly because the SWIS generates around 70% of its power from fossil fuels (coal and gas) with the remaining share generated by renewables (wind, solar, and landfill gas) [63]. 

Table 6 presents the main results of the current study and those of previous LCAs assessing hemp-based construction materials. Although the reviewed studies considered the same FU (1 m^2^ of material), a direct comparison with their results was not possible due to the variation in the composition of the hemp-based materials assessed, i.e., hurds-to-binder ratio, type of binder, additional components (timber, mortar, render), and varying system boundaries across the studies. However, it is useful to contextualise this study’s results within the wide range of outcomes in the existing literature.

Most of the studies found that hemp-based materials exhibit carbon sequestration potential, i.e., a negative CF, when the biogenic uptake is considered. In addition, the studies confirmed that synthetic fertilisers and binders are usually the hotspots as their production is energy intensive and usually powered by fossil fuels. Accordingly, a sensitivity analysis was performed to examine the effects of varying the main energy source during post-farm on the CF of the hemp-based board.

**Table 6:** CF expressed in kg CO_2_ eq per m^2^ of hemp-based construction materials reported in existing LCA studies.

Location of the Study	Hemp-Based Construction Material	System Boundary	Binder	Hotspot	CF kg CO_2_ eq/m^2^	Ref.
The existing literature						
UK	Lime–hemp concrete	Cradle to gate	Lime	Binder production	−36.08	[21]
France	Lime–hemp concrete	Cradle to grave	Lime	Binder production	−0.016	[29]
Italy	Lime–hemp concrete blocks	Cradle to use	Lime	Binder production	−12.09	[30]
Latvia	Lime–hemp concrete	Cradle to gate	Lime	Fertiliser and leaching	−19.28 to 4.88	[31]
	Magnesium–hemp concrete		Magnesium oxychloride	Fertiliser and leaching	−12.68 to 54.29	
Serbia	Lime–hemp concrete	Cradle to grave	Lime	Binder production	−9.696 to 14.899	[18]
Current study						
Australia	Hemp board	Cradle to gate	Bio-based binder	Binder production	−2.302	

#### 3.2.3. Sensitivity Analysis

A sensitivity analysis was conducted by replacing WA’s public grid (SWIS) with electricity generated by solar photovoltaics (PV) in three different proportions: 25%, 50%, and 100%. The analysis reported that the CF of hemp-based boards was reduced by 41%, 82%, and 164%, respectively (Table 7). These results show that the introduction of solar power to replace the public mix partially or totally during post-farm activities can substantially mitigate the CF of hemp-based boards. Moreover, the CO_2_ uptake significantly outweighs total CO_2_eq emissions when a large proportion of the SWIS power is replaced.

These results can encourage board manufacturers to install their own solar PV systems and obtain larger carbon gains as WA presents high potential to generate solar power [64]. However, further research is needed to evaluate whether the installation of PV systems is financially viable.

### 3.3. Comparison with Traditional Materials

**Figure 5:**
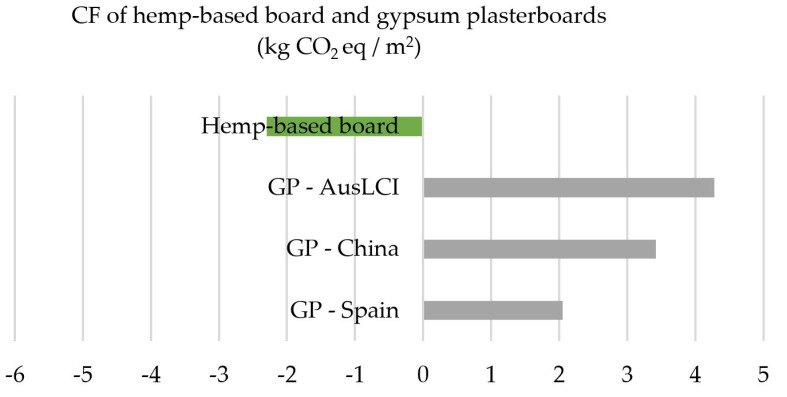
CF of the production of different boards (kg CO_2_ eq/m^2^).

The environmental gain resulting from the use of hemp hurds as feedstock for boards was assessed through the comparison with traditional gypsum plasterboards (here after referred to as GP) as scholars have suggested that hemp-based materials are suitable to replace them [38]. Figure 5 compares the results of this study with those of previous research, assessing the CF of 1 m^2^ of GP. Rivero, Sathre [40] reported that the GHG emissions from GP production in Spain was equivalent to 2.05 kg CO_2_ eq/m^2^. More recently, Zhang, Ma [65] calculated that the CF of the production of phase change GP was 5.6 kg CO_2_ eq/m^2^ in China. Similarly, the AusLCI reported that 9.94 kg of GP (which corresponds to 1 m^2^ of GP) emits 4.28 kg CO_2_ eq.

Table 8 illustrates the potential reduction in embodied carbon emissions (kg CO_2_ eq/m^2^ of usable floor area) for using hemp-based boards as a replacement for traditional GP in different Australian buildings archetypes (residential, commercial, and industrial). The results suggest that hemp-based boards have environmental advantage over traditional materials in terms of global warming. This is primarily due to the biogenic sequestration of hemp during crop growth.

Despite environmental benefits suggested in this and previous studies, the use of hemp-based construction materials is limited in Australia. Moreover, conventional materials such as GPs are widely used nationally due to the availability of gypsum, low price, and relatively ease of manufacture and workmanship [13,68]. Therefore, it is necessary to explore and compare economic and social implications of hemp-based boards with traditional materials. This could be the objective of future research which can help understand the sustainability implications of hemp as feedstock for building materials.

## 4. Conclusions

This study has applied the ISO 14040:2006 LCA methodology to calculate the carbon footprint (CF) (expressed as CO_2_ eq emissions) of hemp-based boards developed in Western Australia (WA), composed of hemp hurds and a bio-based binder. The functional unit was 1 m^2^ of hemp-based board, with a system boundary from cradle to gate, i.e., pre-farm, on-farm, and post-farm activities. The CF of hemp-based boards was divided into two distinct phases: the CF of hemp hurds, i.e., from paddock to hurds, and the CF of hemp-based boards, i.e., from paddock to board.

The CF of 1 kg of hemp hurds produced in WA’s South-West was estimated as −1.031 kg CO_2_ eq, and it was 1.37 kg CO_2_ eq/kg when accounting for biogenic uptake. The primary contributor to carbon emissions was the production of urea (21%), followed by the decortication process (21%). The study demonstrated that several factors could influence the CF of hurds, including site-specific parameters (i.e., yield, agricultural inputs requirements, and biogenic uptake), as well as methodological aspects (i.e., the choice of the allocation method and soil emission factors). Sensitivity analyses reported that using a regional emission factor when estimating direct N_2_O emissions reduced the CF by 4%, compared with that calculated using a default value, whereas the use of the economic allocation method reduced the CF by 9%.

The CF of 1 m^2^ of hemp-based board was estimated to be −2.302 kg CO_2_ eq, including carbon uptake. Electricity sourced from the public grid for bio-based binder production during the post-farm stage constituted 26% of the primary carbon pool, followed by urea production (14%) during the pre-farm stage. Overall, the use of electricity from the SWIS (South West Interconnected System) during the post-farm stage contributed to 45% of the total emissions. Sensitivity analysis revealed that the CF of hemp-based boards was highly sensitive to the energy source; for instance, a complete shift from the SWIS to solar power reduced the CF from −2.30 to −6.07 kg CO_2_ eq (a change of 164%).

The results suggest that hemp-based boards exhibit significantly lower embodied greenhouse gas (GHG) emissions compared to conventional materials, such as gypsum plasterboards, mainly due to the biogenic carbon sequestered during plant growth. Future research should assess the economic and social implications of hemp-based boards from an Australian context to ascertain if they could be a sustainable alternative to traditional materials.

## Figures and Tables

**Table 2:** CF in kg CO_2_ eq per m^2^ of LHC reported in previous LCA studies.

Location of the Study	U-Value W/m^2^K	Thermal Conductivity W/mK	Hotspot	CF kg CO_2_ eq/m^2^ LHC	Reference
United Kingdom	0.19	0.057	Lime production	−36.08	[21]
France	0.36	0.086	Lime production	−0.016	[29]
Italy	0.27	N.D.	Lime production	−12.09	[30]
Serbia	0.30	0.0894	Lime production	−9.69 to 14.89	[18]

**Table 3:** Inventory inputs to produce 1 m^2^ of hemp-based board (FU), sources, and collection method.

Inputs	Quantity	Unit	Source and Collection Method
*Pre-farm*			
*Materials*			
Seeds	1.55 × 10^−2^	kg/FU	FFLI and DPIRD; interviews and questionnaires
Urea	1.03 × 10^−1^	kg/FU	
Potassium sulphate	6.18 × 10^−2^	kg/FU	
Monoammonium phosphate (MAP)	1.03 × 10^−1^	kg/FU	
Glyphosate	8.76 × 10^−4^	L/FU	
*Transport from manufacturer to paddock*			
Small truck	1.10 × 10^−2^	Tkm */FU	FFLI and DPIRD; interviews and questionnaires/assumptions (see Table 4)
Freight ship	4.88 × 10^−1^	tkm/FU	
Articulated truck	2.34 × 10^−1^	tkm/FU	
*On-farm*			
Ripper	3.09 × 10^−3^	ha/FU	FFLI; interviews and questionnaires/technical specifications (tractor: John Deere 9R 390 and associated attachments)
Seeder	3.87 × 10^−3^	ha/FU	
Sprayer (weed control)	6.87 × 10^−4^	ha/FU	
Sprayer (fertilisation)	9.16 × 10^−4^	ha/FU	
Harvester	2.21 × 10^−3^	ha/FU	Technical specifications (hemp cutter Laumetris KP-4)
Harrowing	3.63 × 10^−4^	ha/FU	FFLI; interviews and questionnaires/technical specifications (tractor: John Deere 9R 390 and associated implements)
Baler	3.09 × 10^−3^	ha/FU	
Irrigation	4.85 × 10^−3^	ha/FU	DPIRD Report [50]
*Transport from paddock to board manufacturer*			
Articulated truck	1.25	tkm/FU	FFLI and MIRRECO^®^; interviews and questionnaires
*Post-farm*			
*Indoor transportation (diesel use)*			
Forklift	1.04 × 10^−3^	L/FU	Technical specifications (Hyster H2.0XT)
*Electricity use*			
Decorticator	4.68 × 10^−1^	kw/FU	Technical specifications (HempTrain™)
Mixer	3.54 × 10^−3^	kw/FU	Technical specifications (Nasser Machinery)
Bio-based binder	3.30	kw/FU	MIRRECO^®^; interviews and questionnaires/Literature review [51]
Presser (boiler)	1.17 × 10^−1^	kw/FU	MIRRECO^®^; technical specifications (Italpresse Model XL/10 38-16 PMBO Hydraulic Hot Press)
Presser (hydraulic pump)	9.77 × 10^−3^	kw/FU	

* tkm = tonne km travelled.

**Table 7:** CF expressed in kg CO_2_ eq per m^2^ of hemp-based construction materials reported in existing LCA studies.

Sensitivity Analysis	Emissions (kg CO_2_ eq/m^2^)	Uptake (kgCO_2_/m^2^)	CF (kg CO_2_ eq/m^2^)	Percentage of Reduction
Baseline-SWIS	8.29	−10.59	−2.30	Baseline
Solar energy, 25%	7.34	−10.59	−3.25	41%
Solar energy, 50%	6.40	−10.59	−4.19	82%
Solar energy, 100%	4.52	−10.59	−6.07	164%

**Table 8:** Potential reduction in embodied carbon emissions of replacing gypsum plasterboards with hemp-based boards per m^2^ of usable floor area (UFA) for different building archetypes in Australia.

Building Archetypes	Material Intensity * (kg/m^2^ of UFA)	Material Use ** (m^2^/m^2^ of UFA)	GWP *** (kg CO_2_ eq/m^2^ of UFA)		Carbon Reduction Potential **** (kg CO_2_ eq/ m^2^ of UFA)
	Plasterboard	Plasterboard	Plasterboard	Hemp-Based Board	
Residential					
Single house	28.8	2.90	10.43	−6.67	17.10
Semi-detached house	28.3	2.85	10.25	−6.55	16.80
1- and 2-storey apartment	19.8	1.99	7.17	−4.58	11.75
3-storey apartment	14.5	1.46	5.25	−3.36	8.61
Apartment with 4 or more storeys	7.1	0.71	2.57	−1.64	4.21
Commercial					
1–3-storey commercial	13.2	1.33	4.78	−3.06	7.84
4–7-storey commercial	5.7	0.57	2.06	−1.32	3.38
8–35 storey commercial	4.4	0.44	1.59	−1.02	2.61
Industrial					
1-storey industrial	22.6	2.27	8.18	−5.23	13.42
2-storey industrial	11.3	1.14	4.09	−2.62	6.71

* Material intensity of Australian buildings from Soonsawad, Martinez [66]. ** Material use estimated by dividing material intensity with the density of gypsum plasterboards (around 9.94 kg/m^2^) in Australia [67]. *** GWP: global warming potential (kg CO_2_ eq/m^2^ of UFA) estimated via multiplying material consumption with CF of plasterboard (4.28 kg CO_2_ eq/m^2^) and CF of hemp-based board (−2.302 kg CO_2_ eq). **** Carbon reduction potential: GWP plasterboard−GWP hemp-based board.

## Data Availability

Data are contained within the article.
